# Turkish adaptation of the CTONI-2: psychometric properties and student performance profiles

**DOI:** 10.3389/fpsyg.2026.1876351

**Published:** 2026-09-08

**Authors:** Burhanettin Ozdemir, Nesrin Seef, Burcu Atar

**Affiliations:** 1 College of Business, Alfaisal University, Riyadh, Saudi Arabia; 2 Gaziantep Universitesi, Gaziantep, Türkiye; 3 Hacettepe Universitesi, Ankara, Türkiye

**Keywords:** CFA, CTONI-2, nonverbal intelligence test, student performance, test adaptation

## Abstract

**Introduction:**

The Comprehensive Test of Nonverbal Intelligence-2 (CTONI-2) assesses nonverbal intelligence from ages 6–90 with 6 subtests divided into a Pictorial composite and a Geometric composite. There was no previously validated Turkish version. The purpose of this study was to adapt the CTONI-2 for Turkish primary-school children aged 8–10 and to test its reliability and validity, while it was also applied to a small pilot sample of secondary-school children (*N* = 54) to examine performance levels on subtests and preliminary group differences by gender, grade level, and age.

**Methods:**

The participants of the adaptation study were 590 primary-school students in Turkey. Internal-structure evidence was examined using CFA, and convergent validity was examined through correlations with Raven's Standard Progressive Matrices. KR-20 and test-retest techniques were employed to assess reliability. All six subtests were then administered to the pilot secondary-school sample. Patterns of performance were explored using independent-samples t-tests, one-way ANOVAs, and Pearson correlations, with raw-score percentages used only for exploratory descriptive interpretation.

**Results:**

CFA results showed a unidimensional structure with CFI = 0.95, RMSEA = 0.07 and CR = 0.78. KR-20 ranged from 0.79 to 0.94; test-retest from 0.81 to 0.96. The pilot application scores were between 64 and 72% of maximum score per subtest, with all means in the Average-to-Above-Average range. There were no significant differences between males and females. Grade 10 students performed better than Grade 11 students on Geometric Analogies (*d* = 0.83), and age was negatively correlated with geometric reasoning subtests (*r* = −0.28 to −0.33).

**Discussion:**

Given the small and unevenly distributed pilot sample, these findings should be interpreted cautiously. The results support the reliability and validity of the Turkish CTONI-2 in the primary-school adaptation sample and provide preliminary application evidence for secondary-school students.

## 1 Introduction

The origins of intelligence testing lie in the late 19th century, and it has been defined by a long-standing attempt to measure mental ability in a scientifically valid and culturally fair way. The earliest systematic intelligence assessment on record dates to the Chinese imperial civil-service examinations of 2200 BC, while in ancient Egypt competency testing informed administrative selection—indicating that societies have long sought objective criteria for evaluating capability (Heiser, 2023; Sternberg, 2019). The formal psychometric tradition began in Europe with Galton's anthropometric laboratory and came to a formalization in the age-graded scales of Binet and Simon (1905) and the concept of the intelligence quotient (IQ) formalized by Stern.

Modern intelligence theory recognizes that cognitive ability includes several domains, including verbal, numerical, spatial, and figural reasoning. Nonverbal intelligence, defined as the ability to reason with visual-spatial and figural information with reduced dependence on language, has therefore received increasing attention in educational and psychological assessment (Euler et al., 2023). Nonverbal assessments are particularly pertinent when language proficiency or verbal comprehension, or cultural familiarity, may affect test performance (Carroll et al., 2022). While they are sometimes suggested for certain groups where language is a primary assessment issue such as children with hearing impairment, individuals who are autistic and have difficulties with verbal communication, and students from migrant or linguistically diverse backgrounds, these are all contextual motivations or applications, but are not assessed in the present study (Berube, 2021; Hasnain et al., 2023; Rogers and Tazi, 2025). The main interest of the present study is the adaptation of a language reduced nonverbal reasoning test which is conducted in the target educational population to be culturally appropriate, familiar to the students and psychometrically valid.

The CTONI-2 (Hammill et al., 2009) is a nonverbal intelligence test for ages 6 to 90 which is composed of six subtests, two composites, the Pictorial Composite (analogies, classification, and sequences with meaningful pictures) and the Geometric Composite (analogies, categories, and sequences with abstract figures). By separating pictorial from geometric content, the instrument permits differentiated profiling of concrete visual reasoning and abstract figural reasoning. Although the original CTONI-2 covers a broad age range, the present Turkish adaptation was intentionally conducted as an age-specific first-stage validation rather than a full national standardization across all eligible ages. The initial adaptation focused on primary-school children aged 8 to 10 because this age band is developmentally appropriate for evaluating item comprehensibility, visual-cultural familiarity, and basic response processes in school-based administration. Before the present study, no validated Turkish version of the CTONI-2 was available for school-aged children, limiting its use in Turkish educational assessment and research.

The aim of the first part of this paper is to adapt and psychometrically validate the Turkish version of the CTONI-2 in a primary-school sample aged 8 to 10 years, where linguistic, cultural, and developmental appropriateness of the adapted items could be examined under controlled school-based conditions. The second part applies the adapted instrument to a separate secondary-school sample aged 14 to 17 years to examine subtest performance profiles and demographic group differences. This second stage was not designed as adolescent norm development, but as an initial application of the adapted instrument in an older school group to explore its descriptive utility beyond the primary-school validation sample.

### 1.1 Literature review

Nonverbal intelligence assessment has particular relevance for school-aged children because educational decisions often require information about reasoning ability that is less dependent on verbal expression, acquired vocabulary, or language-specific test demands. In school settings, nonverbal measures can support the evaluation of visual-spatial reasoning, abstract reasoning, and problem-solving skills while reducing the influence of linguistic load. This is especially important in test adaptation because language-reduced instruments may still include culturally familiar images, visual conventions, or response expectations that require empirical evaluation in the target educational population.

The existing evidence suggests that the CTONI-2 may yield some psychometric information, though the utility of the instrument must be considered in the context of the culture and education where it is implemented. The scores of the CTONI-2 have been found to be reliable in clinical assessment (Grondhuis, 2020) and differences in scores by age, gender, and parent education level were observed with children in Lebanon (Summaka et al., 2024). Additionally, even measures of language that were reduced, such as receptive language, also contributed to the variance of the nonverbal IQ scores (Short, 2021). These results support local validation of the instrument, not the idea that a nonverbal instrument translates equally across languages, school systems and cultural contexts.

In addition to the cost of importing and re-standardizing measures from abroad, there is a cost of research funds and practitioner resources that could otherwise be used to support indigenous measures. Even with this limitation, several widely used international nonverbal instruments have been used in clinical research and educational research with the Turkish researchers who have conducted validity and reliability studies (Acar et al., 2019; Biçer and Sari, 2017; Karakelle, 2012; Şahin, 2014; Bildiren et al., 2017). Of this work, (Şahin and Düzen 1994) published a standardization of the Raven Standard Progressive Matrices Test for children aged 6–15, and later others added other normative samples into the BILNOT Battery.

Turkish research has also provided important evidence for nonverbal cognitive assessment. Several international nonverbal instruments have been used in Turkish clinical and educational research, including studies by Acar et al. (2019) ), Biçer and Sari (2017), Karakelle (2012), and Şahin (2014). Şahin and Düzen (1994) standardized the Raven Standard Progressive Matrices Test for children aged 6 to 15, Korkmaz et al. (2018) standardized the TONI-3 for children aged 6 to 11, and Bildiren et al. (2021) examined the reliability and validity of the Colored Progressive Matrices Test for children aged 4 to 6. The feasibility of nonverbal cognitive assessment was shown in Turkish children in these studies, but no validity and reliability evidence was found for the CTONI-2.

The remaining gap is therefore specific rather than general: there is no validated Turkish version of the CTONI-2 for school-aged children. This is important because the CTONI-2 is unique in using both pictorial and geometric subtests and provides the option for composite level interpretation. Furthermore, there is also lack of uniformity across the previous Turkish studies in terms of sampling and representativeness which prevents generalizing the results of the other instruments to the CTONI-2. The present study addresses this gap by examining cultural item adaptation, construct validity, criterion-related validity, internal consistency, and test-retest reliability in Turkish primary-school children, followed by a preliminary pilot application with secondary-school students.

## 2 Methodology

This study used a cross-sectional survey design with two sequential stages. The first stage involved the cultural adaptation and psychometric validation of the Turkish version of the CTONI-2 with primary-school students aged 8 to 10 years. The second stage involved an exploratory application of the adapted instrument with a separate secondary-school sample aged 14 to 17 years. The primary-school sample was used to examine item appropriateness, construct validity, criterion-related validity, internal consistency, and test-retest reliability. The secondary-school sample was used only to describe preliminary score profiles and group differences in an older school group and was not intended to provide adolescent norms or full age-range validation.

### 2.1 Research procedure

The research process followed a chronological adaptation and validation sequence. First, all 150 CTONI-2 items were reviewed for cultural appropriateness by an expert panel consisting of eight members: four measurement and evaluation specialists, two psychological counselors, and two classroom teachers. The review focused on whether the pictorial and geometric content was understandable, culturally appropriate, developmentally suitable, and relevant for Turkish school-aged children. Experts rated each item using a four-point relevance and appropriateness scale. Item-level content validity indices (I-CVI) were calculated as the proportion of experts rating an item as either 3 or 4. The minimum acceptable I-CVI was set at 0.78, following Polit and Beck's criterion for expert-panel agreement (Polit and Beck, 2006). Based on this expert review, four items in Pictorial Analogies, two items in Pictorial Sequences, and one item in Photographic Classification were identified as requiring cultural modification. No CTONI-2 items were deleted. No changes were required for the Geometric subtests.

Second, a first pilot administration was conducted with 90 primary-school students to examine item comprehensibility, clarity of visual content, and cultural familiarity of the modified items. During this phase, the researchers monitored whether students understood the task requirements, whether any pictorial content created confusion, and whether administration procedures were appropriate for the target age group. Third, a second pilot administration was carried out with 60 students to ensure that the revised items and administration procedures performed well prior to the main validation administration. The pilot results were used to ensure the suitability of the item changes based on experts and to determine if additional changes are required. No modification of the Geometric subtests was made. Fourth, the adapted form was administered to the main primary-school validation sample. In a subsample of 100 students, we checked the convergent validity with Raven's Standard Progressive Matrices. Test-retest reliability was checked 3 weeks later on 75 students from the main sample that was administered and were able to be re-administered in the same school context. The same Turkish version of the CTONI-2 form was given at both time points and all six subtests were re-administered. The retest administration was administered according to the same standardized test instructions, item order, scoring procedures and school-based testing conditions as the first administration.

### 2.2 Adaptation and validation sample

The adaptation and validation sample consisted of primary-school students aged 8 to 10 years from two schools in Ankara during the 2016 to 2018 school years. This age range was selected because children in this developmental period can complete all six CTONI-2 subtests independently while still being sensitive to potential cultural unfamiliarity, visual ambiguity, or item comprehension difficulties that may be less visible in older students. This sample formed the basis for the main psychometric analyses of the Turkish adaptation.

### 2.3 Secondary-school application sample

After the adaptation stage, the Turkish CTONI-2 was administered to a separate secondary-school application sample. This sample included 54 students aged 14 to 17 years (*M* = 15.89, SD = 0.79), comprising 38 female students (70.4%) and 16 male students (29.6%). Students were enrolled in Grade 10 (*n* = 41, 75.9%) and Grade 11 (*n* = 13, 24.1%). This stage was included to describe adolescent performance profiles and examine preliminary differences by gender, grade level, and age. Because the sample was small and unevenly distributed across grades, the findings from this stage should be interpreted as exploratory pilot evidence rather than as adolescent validation or norming evidence.

### 2.4 Data collection and ethical procedures

Data collection was conducted in school settings after the required institutional and school permissions had been obtained. Schools were recruited using school-based permission. The procedures were followed and eligible students were invited to participate within the age groups indicated. For the adaptation/validation and application stages. Participation was voluntary. Parental consent and student assent were obtained before data collection, and students' responses were analyzed anonymously to protect confidentiality.

The CTONI-2 was administered by trained research personnel/administrators familiar with the standard administration procedures of the instrument. Testing was conducted in designated school rooms under controlled conditions, and students completed the subtests according to the CTONI-2 administration guidelines. The CTONI-2 was administered in small groups in designated school rooms under standardized conditions. The same standardized instructions, item order, scoring procedures, and testing conditions were followed across administrations to support procedural consistency and replicability.

### 2.5 Data analysis strategy

Confirmatory factor analysis was employed to explore the construct validity. To assess convergent validity, the scores of the CTONI-2 were correlated with the Raven's Standard Progressive Matrices in the convergent-validity subsample. The internal consistency was assessed by KR-20 coefficient. Pearson test-retest correlation coefficients were computed for each subtest and overall score between the 3-week span to assess temporal stability.

For the secondary school application sample, descriptive statistics were obtained for all subtests and composite scores. Gender and grade-level comparisons were conducted using independent-samples tests and Cohen's d (Cohen, 1988) was calculated as an effect-size estimate. The parametric results were interpreted with assumptions (due to unequal group sizes of the grade levels examined). To determine whether the scores were normally distributed, the Shapiro-Wilk tests were used, and the score distributions were inspected. To determine whether there was homogeneity of variance between the scores, Levene's test was employed. For the cases in which assumptions were not entirely met or where group sizes were significantly different, sensitivity analysis was conducted using Welch's *t*-test and Mann–Whitney U tests. To decrease the risk of Type I errors in multiple comparisons, Holm-adjusted *p*-values were analyzed. One-way ANOVA examined age-group effects, and Pearson correlations assessed age-related and intercorrelation patterns. These raw scores were converted to percentages of the maximum possible score and compared to benchmarks reported by Hammill et al. (2009) for an exploratory descriptive profile of the secondary-school pilot sample. All analyses were conducted using R (version 4.4.2; R Core Team, 2024).

## 3 Findings

### 3.1 Participant characteristics

The primary-school adaptation and validation stage included students aged 8 to 10 years from two primary schools in Ankara. As shown in Table 1, the first pilot included 90 students, the second pilot included 60 students, and the main validation administration included 500 students. In the main validation administration, 407 students (81.4%) were from Turkish Primary School 1 and 93 students (18.6%) were from Turkish Primary School 2. The available demographic and educational information for the primary-school sample was limited to age range, school, and administration phase.

**Table 1:** Distribution of students across schools.

Province	School	Pilot 1 *n*	Pilot 1 %	Pilot 2 *n*	Pilot 2 %	Main *n*	Main %
Ankara	Turkish primary school 1	60	66.7%	30	50.0%	407	81.4%
Ankara	Turkish primary school 2	30	33.3%	30	50.0%	93	18.6%
Total		**90**	**100%**	**60**	**100%**	**500**	**100%**

Pilot 2 students were included in the final application group (*N* = 500 total).

The secondary-school application sample included 54 students aged 14 to 17 years (*M* = 15.89, SD = 0.79). The sample comprised 38 female students (70.4%) and 16 male students (29.6%). Students were enrolled in Grade 10 (*n* = 41, 75.9%) and Grade 11 (*n* = 13, 24.1%). This sample was therefore small and unevenly distributed across grade levels, and its findings should be interpreted as exploratory pilot evidence.

Information on socioeconomic status, parental education, clinical or developmental diagnoses, language background, hearing status, and special educational needs was not collected. As a result, representativeness with respect to these characteristics could not be evaluated, and the findings should not be generalized to clinical, hearing-impaired, language-disordered, autistic, migrant, or special-education populations.

### 3.2 Validity evidence

#### 3.2.1 Content validity

Based on expert consultation, seven items were identified as culturally misaligned in the Pictorial Analogies (*n* = 4), Pictorial Sequences (*n* = 2), and Photographic Classification (*n* = 1). These items were modified to improve cultural familiarity and visual interpretability for Turkish school-aged children, while preserving the reasoning demand of the original item. No items were removed from the adapted form. After revision, all 150 items met the item-level content validity criterion, with I-CVI values greater than 0.78. Scale-level CVI was not used as the primary decision criterion; the content-validity decision was made at the item level because the purpose of the review was to identify culturally problematic stimuli requiring revision. The modified items are summarized in Table 2. Because the CTONI-2 is a copyrighted test, the table reports the nature and rationale of each modification without reproducing the original stimuli.

**Table 2:** Summary of culturally modified CTONI-2 items.

Subtest	Modified items	Cultural issue	Revision focus	Post-revision result
Pictorial analogies	4	Culturally less familiar pictorial content	Visual elements revised while preserving analogy reasoning	I-CVI >0.78
Pictorial sequences	2	Ambiguous or culturally less familiar sequence content	Sequence visuals clarified while preserving ordering logic	I-CVI >0.78
Photographic classification	1	Culturally less familiar photographic content	Image content revised to improve recognizability	I-CVI >0.78
Geometric subtests	0	No cultural issue identified	No revision required	Retained unchanged

#### 3.2.2 Internal structure evidence

**Figure 1:**
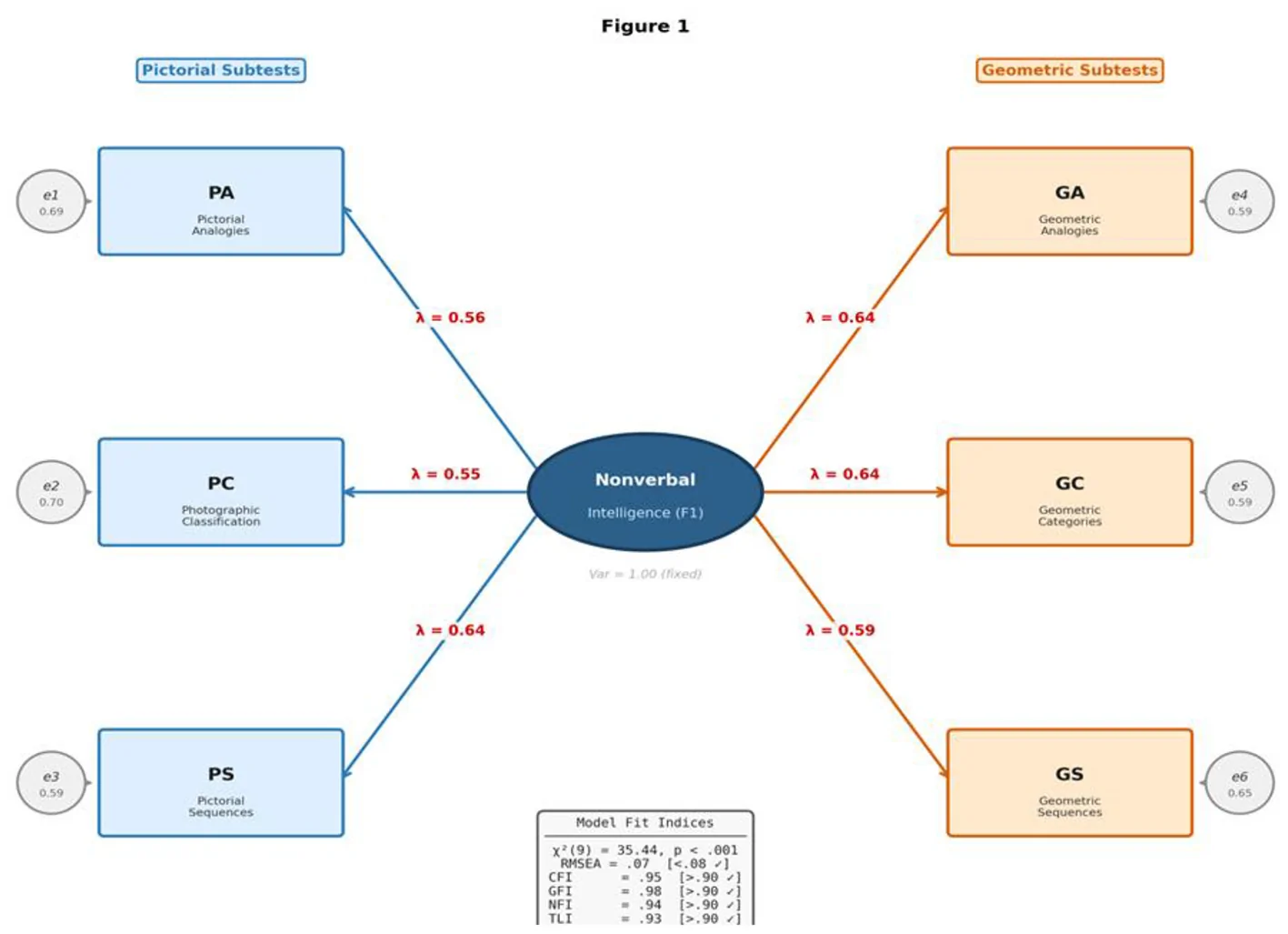
Path diagram for the single-factor CFA model of the Turkish CTONI-2. Ellipse = latent factor (F1, Nonverbal Intelligence; variance fixed to 1.00). Rectangles = observed subtest scores. Circles = error terms (values = error variances = 1 – λ^2^). Values on paths are standardized factor loadings (λ). Blue arrows = Pictorial subtests; Orange arrows = Geometric subtests. Model fit indices are embedded in the lower panel.

A one-factor model in which all six observed subtests loaded onto one latent Nonverbal Intelligence factor was examined using confirmatory factor analysis (CFA). CFA was used to test the hypothesized structure derived from the original CTONI-2 framework rather than to explore a new dimensional structure. Because this is the first Turkish validation of the CTONI-2, the CFA findings should be interpreted as evidence for the expected structure in the present primary-school sample, not as evidence that alternative structures are ruled out. Future studies should examine alternative factor structures using exploratory and confirmatory approaches, preferably with split-sample designs and larger, more diverse Turkish samples. Table 3 shows the standardized factor loadings, explained variance (R^2^), error variance, and error terms. The path diagram is presented in Figure 1.

**Table 3:** Standardized factor loadings, explained variance, and error terms for the single-factor CFA model.

Subtest	λ (Std.)	*R*^2^ (λ^2^)	Error var.	Error term
Pictorial analogies (PA)	0.56	0.31	0.69	e1
Geometric analogies (GA)	0.64	0.41	0.59	e_2_
Photographic classification (PC)	0.55	0.30	0.70	e3
Geometric categories (GC)	0.64	0.41	0.59	e4
Pictorial sequences (PS)	0.64	0.41	0.59	e5
Geometric sequences (GS)	0.59	0.35	0.65	e6
**Average/total**	**0.61**	**0.37 (AVE)**	**0.62 (avg)**	**CR** **=** **0.78**

λ, standardized factor loading; *R*^2^, proportion of variance explained by the latent factor; Error Var., 1 – λ^2^; AVE, average variance extracted; CR, composite reliability. All loadings significant at *p* < 0.001.

Standardized loadings ranged from 0.55 for Photographic Classification to 0.64 for Geometric Analogies, Geometric Categories, and Pictorial Sequences. All loadings exceeded the conventional minimum of 0.40 and the more stringent 0.50 criterion. The R^2^ values ranged from 0.30 to 0.41, indicating that the common latent factor explained a moderate proportion of variance in each subtest. As shown in Table 4, the chi-square ratio was above the preferred threshold, χ^2^/df = 3.94, but the remaining model-fit indices supported acceptable fit: CFI = 0.95, GFI = 0.98, TLI = 0.93, NFI = 0.94, and RMSEA = 0.07. These results provide support for the internal structure of the Turkish CTONI-2, while also indicating that subtest-specific variance remains present.

**Table 4:** CFA model fit indices evaluated against commonly used criteria.

Index	Criterion	Value
χ^2^/df	≤ 2.0 excellent; ≤ 3.0 acceptable (Kline, 2016)	3.94
RMSEA	<0.05 excellent; 0.05–0.08 acceptable (Browne and Cudeck, 1993)	0.07
CFI	>0.95 excellent; >0.90 acceptable (Hu and Bentler, 1999)	0.95
TLI	>0.95 excellent; >0.90 acceptable (Bentler and Bonett, 1980)	0.93
GFI	>0.95 excellent; >0.90 acceptable (Jöreskog and Sörbom, 1984)	0.98
NFI	>0.90 acceptable (Bentler and Bonett, 1980)	0.94
AVE	≥0.50 (Fornell and Larcker, 1981)	0.37
CR	≥0.70 (Hair et al., 2019)	0.78

χ^2^/df, chi-square divided by degrees of freedom; RMSEA, root mean square error of approximation; CFI, comparative fit index; TLI, Tucker–Lewis index; GFI, goodness-of-fit index; NFI, normed fit index; AVE, average variance extracted; CR, composite reliability.

The Average Variance Extracted value (AVE = 0.37) was lower than the 0.50 threshold proposed by Fornell and Larcker (1981), suggesting that the common latent factor explained less than 50% of the average variance in the six observed subtest scores. This result indicates that while there is meaningful common variance among the subtests, there is also significant subtest-specific or unexplained variance in the scores. While this result is consistent with the content-heterogeneous structure of the CTONI-2 that includes both pictorial and geometric reasoning tasks, it should be viewed as a weak form of convergent evidence because it is not sufficient to conclude that the tests are linked. Thus, Parkin et al. (2017) is referred to only as evidence that CTONI-2 subtests may exhibit differentiated psychometric behavior in particular samples and not as evidence that low AVE values are characteristic of intelligence tests as a general class of tests. The internal coherence of the latent construct was satisfactory (CR = 0.78). The low AVE, however, suggests a caution in interpreting the unidimensional model, and future research could explore other factor structures and age-specific measurement properties.

#### 3.2.3 Convergent validity evidence

Pearson correlations between CTONI-2 scores and Raven's Standard Progressive Matrices were examined as convergent validity evidence because both instruments assess nonverbal reasoning. The results are presented in Table 5. All coefficients were statistically significant at *p* < 0.001.

**Table 5:** Correlation coefficients between CTONI-2 scores and Raven's SPM.

Test	Total CTONI-2	Pictorial composite	Geometric composite
Raven SPM (total)	0.54^*^	0.48^*^	0.52^*^

SPM, standard progressive matrices. ^*^p < 0.001. *N* = 100.

The coefficients ranged from 0.48 to 0.54, indicating moderate convergence between the Turkish CTONI-2 and Raven's SPM. This level of association is theoretically appropriate because both instruments assess nonverbal reasoning, but they differ in item format, subtest composition, and content domains. The moderate correlations are also consistent with Parkin et al. (2017), who reported correlations between CTONI-2 composites and external measures within a comparable range.

### 3.3 Reliability study

KR-20 internal consistency and test-retest reliability coefficients are presented in Table 6.

**Table 6:** KR-20 and test-retest reliability coefficients for CTONI-2 subtests and composites.

Test/subtest	KR-20	Test-retest *r*
Pictorial analogies	0.90	0.94
Photographic classification	0.79	0.81
Pictorial sequences	0.79	0.84
Pictorial composite	**0.92**	**0.88**
Geometric analogies	0.89	0.96
Geometric categories	0.87	0.89
Geometric sequences	0.84	0.83
Geometric composite	**0.92**	**0.89**
Total score	**0.94**	**0.88**

KR-20, Kuder–Richardson formula 20; Test-retest interval, 3 weeks (*n* = 75). Composite rows bolded.

The internal consistency of the scales varied from 0.79 to 0.94, with the Total Score having the highest internal consistency (0.94). The test-retest coefficients ranged from 0.81 to 0.96, with the highest being for Geometric Analogies (0.96). All values are at or above 0.70, which is the minimum standard for applied decision-making contexts (Nunnally and Bernstein, 1994) and fall in the typical range of 0.79–0.94 reported for the original CTONI-2 standardization (Hammill et al., 2009).

### 3.4 Application: student performance analysis

Descriptive statistics and exploratory group-comparison analyses for the secondary-school application sample (*N* = 54) are reported in this section. Because this sample was small, unevenly distributed across grade levels, and not designed for adolescent norming, these results should be interpreted as preliminary descriptive evidence only.

#### 3.4.1 Descriptive statistics

Descriptive statistics for all subtests and composites are presented in Table 7. Normality was evaluated using Shapiro-Wilk tests, and most of the data were generally normally distributed (W > 0.90), and with slight negative skewness noted for Geometric Sequences (Skewness = −1.14), due to slight ceiling concentration.

**Table 7:** Descriptive statistics for CTONI-2 subtests and composites (*N* = 54).

Subtest/score	*n*	*M*	SD	Min	Max	Skew	Kurt
Pictorial analogies (PA)	54	16.28	3.42	10	21	−0.44	−0.94
Geometric analogies (GA)	54	17.65	2.64	11	23	−0.24	−0.50
Photographic classification (PC)	54	16.09	3.38	7	21	−0.59	−0.44
Geometric categories (GC)	54	17.89	2.86	10	22	−0.48	−0.17
Pictorial sequences (PS)	54	17.52	2.79	11	23	−0.27	−0.69
Geometric sequences (GS)	54	17.91	3.36	7	23	−1.14	1.43
**Total pictorial**	**54**	**49.89**	**6.62**	**35**	**60**	**−0.57**	**−0.49**
**Total geometric**	**54**	**53.44**	**6.51**	**37**	**65**	**−0.35**	**−0.22**
**Total score**	**54**	**103.33**	**11.59**	**80**	**123**	**−0.32**	**−0.88**

PA, pictorial analogies; GA, geometric analogies; PC, photographic classification; GC, geometric categories; PS, pictorial sequences; GS, geometric sequences. All scores are raw subtest totals.

#### 3.4.2 Descriptive score profile for the secondary-school pilot sample

**Figure 2:**
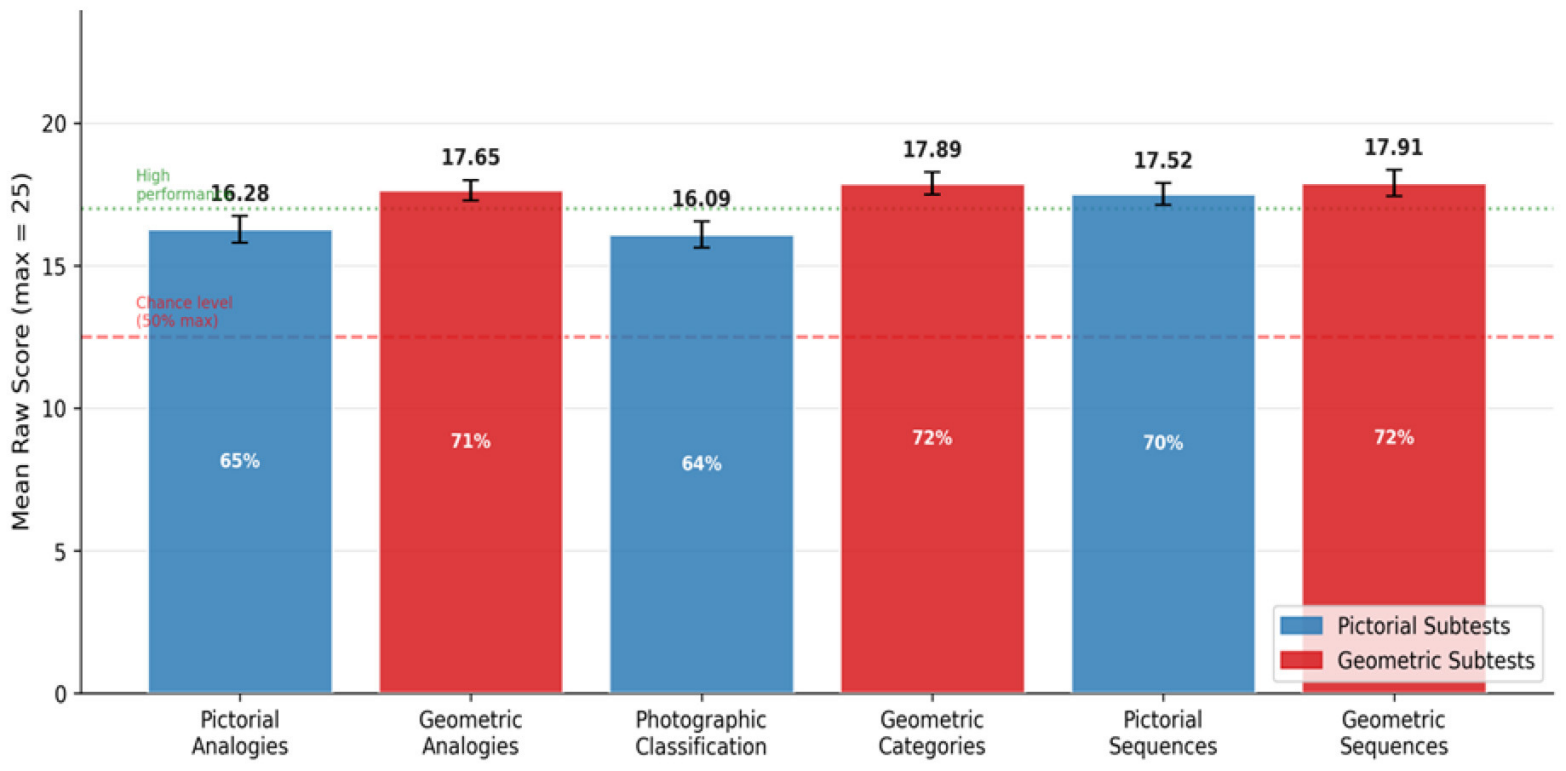
CTONI-2 subtest performance profile with percentage of maximum score (*N* = 54). Bars show mean raw score (max = 25 per subtest). Percentages inside bars indicate % of maximum. Red dashed line = 50% chance level (12.5). Green dotted line = above-average threshold (17.0/25 = 68%). Pictorial subtests in blue; Geometric subtests in red.

For each subtest, raw scores were transformed into percentages of maximum possible (25 items) and classified according to the CTONI-2 descriptive performance bands adapted from Hammill et al. (2009): 0–55% = Below Average; 56–68% = Average; 69–80% = Above Average; >80% = Superior. These percentages were not treated as psychometrically equivalent to the original CTONI-2 age-normed standard-score classifications. Because Turkish age-normed adolescent standard scores were not available and the secondary-school sample was small, the percentage values are presented only for exploratory description and should not be interpreted as normative, diagnostic, or criterion-referenced classifications. The results are provided in Table 8 and Figure 2.

**Table 8:** Descriptive raw-score profile for the secondary-school application sample (*N* = 54).

Subtest	*M*	SD	% of Max	Classification	Interpretation
Pictorial analogies (PA)	16.28	3.42	65.1%	Average	Within expected range; slightly below geometric peers
Geometric analogies (GA)	17.65	2.64	70.6%	Above average	High pictorial-domain analogy; with highest loading (λ = 0.64)
Photographic classification (PC)	16.09	3.38	64.4%	Average	Lowest overall mean; real-image content may introduce cultural variance
Geometric categories (GC)	17.89	2.86	71.6%	Above average	High score; consistent with abstract categorical reasoning ability
Pictorial sequences (PS)	17.52	2.79	70.1%	Above average	Indicates high sequential reasoning with real-world images
Geometric sequences (GS)	17.91	3.36	71.6%	Above average	Equal high mean; key predictor of Total GEO (*r* = 0.91)
**Total pictorial**	**49.89**	**6.62**	**66.5%**	**Average**	Composite in average-to-above range; SD typical for norm groups
**Total geometric**	**53.44**	**6.51**	**71.3%**	**Above average**	Composite above average; geometric domain stronger than pictorial
**Total score**	**103.33**	**11.59**	**68.9%**	**Above average**	Total in average range (SS ≈ 100–108 est.); no floor/ceiling effect

% of Max = mean raw score divided by maximum possible (25 per subtest; 75 per composite; 150 for Total). Classification bands derived from Hammill et al. (2009). SS = estimated standard score based on normative conversion tables for ages 15–17.

All six subtest means fell substantially above the chance level of 12.5 (50% of max), ranging from 16.09 (PC, 64%) to 17.91 (GS, 72%). Four subtests (GA, GC, PS, GS) were in the above moderate range of scores (17.0/25 = 68%) while Pictorial Analogies (65%) and Photographic Classification (64%) were just in the moderate range. The Composite scores for the 49.89/75 (Total Pictorial) and 53.44/75 (Total Geometric) are indicative of a moderate to high range in the ages 15–17 range (based on estimated standard scores; Hammill et al., 2009).

Total Geometric scores were numerically higher than Total Pictorial scores in this sample (71.3% vs. 66.5% of the maximum score). However, because the secondary-school application sample was small and no inferential test was conducted to evaluate the composite-level difference, this pattern should be interpreted only as a descriptive observation. Similarly, Photographic Classification had the lowest mean score among the subtests, but no item-level cultural bias analysis or subgroup invariance analysis was conducted. Therefore, no conclusion is drawn regarding cultural variance in this subtest.

The mean Total Score was 103.33 (SD = 11.59), with observed scores ranging from 80 to 123. These findings suggest that the adapted instrument produced a spread of raw scores in this pilot sample, but they should not be interpreted as evidence of normative performance, adolescent standardization, or generalizable score classification. Larger and more representative Turkish adolescent samples are needed before conclusions can be made about normative standing, subgroup differences, or domain-specific performance patterns.

#### 3.4.3 Gender differences

**Figure 3:**
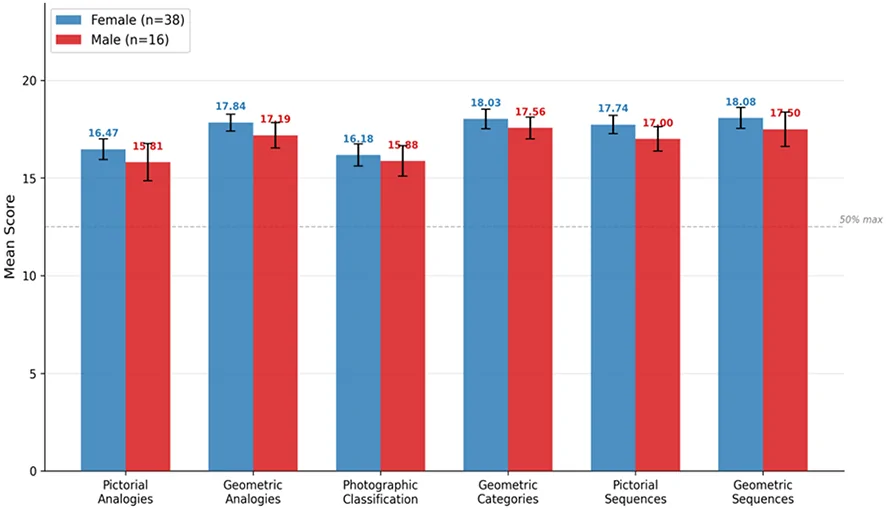
Mean CTONI-2 subtest scores by gender with standard error bars and data labels. Numbers above bars are group means. Error bars = ±1 SE. Dashed line = 50% of maximum (12.5).

**Figure 4:**
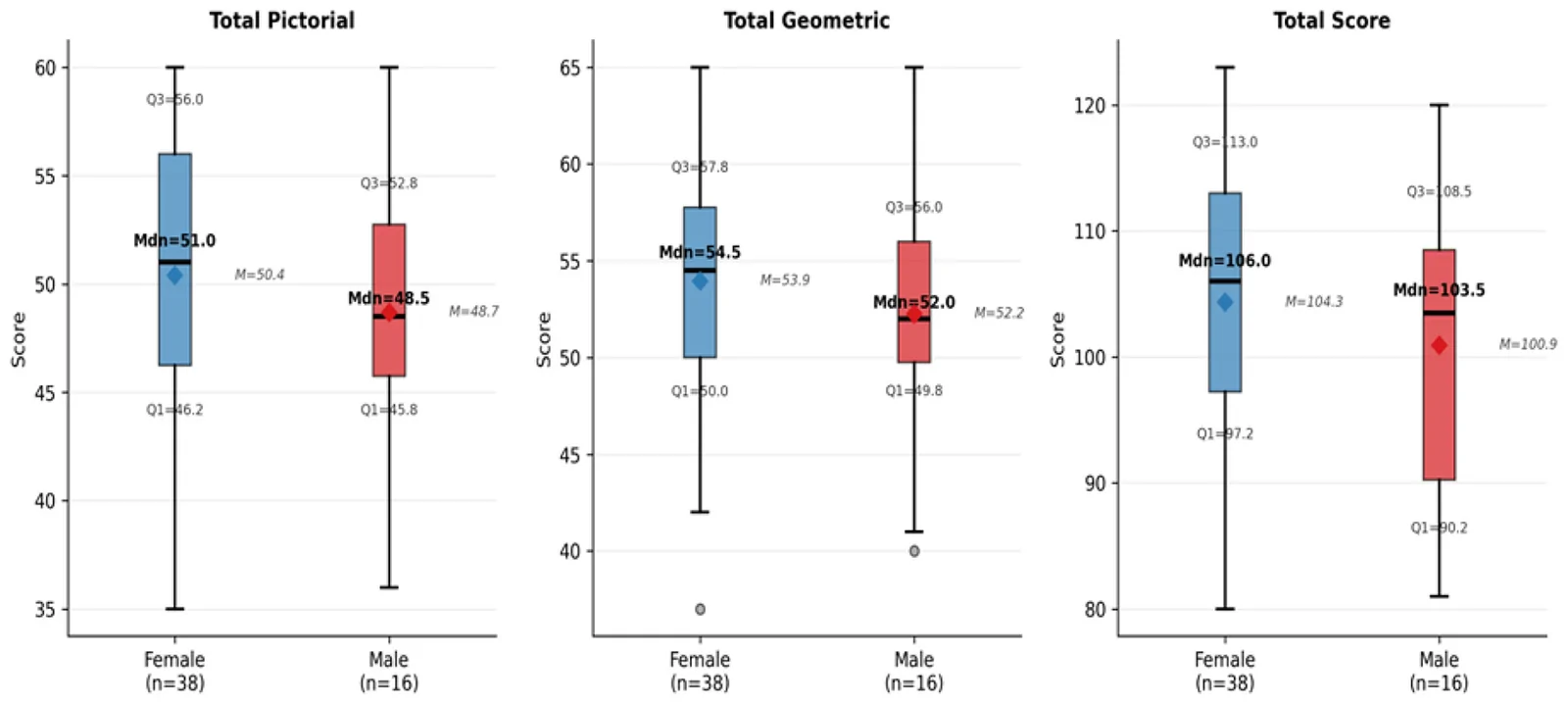
Distribution of total composite scores by gender with descriptive labels. Diamonds = group means. Mdn = median; Q1/Q3 = first/third quartile. Circles = outliers (>1.5 × IQR).

Independent-samples *t*-tests were conducted to compare female (*n* = 38) and male (*n* = 16) students on all subtests and composites. Because the gender groups were small and unequal, these analyses were treated as exploratory. Assumptions were examined before interpreting the parametric comparisons, and Holm-adjusted *p*-values were considered across the multiple subtest and composite comparisons. Results are summarized in Table 9 and Figures 3 and 4.

**Table 9:** Comparison of CTONI-2 scores by gender.

Subtest	F M	F SD	M M	M SD	*d*	*t*	*p*	Sig.
Pictorial analogies	16.47	3.27	15.81	3.82	0.19	0.65	0.521	ns
Geometric analogies	17.84	2.66	17.19	2.61	0.25	0.83	0.410	ns
Photographic classification	16.18	3.51	15.88	3.14	0.09	0.31	0.762	ns
Geometric categories	18.03	3.10	17.56	2.25	0.16	0.54	0.591	ns
Pictorial sequences	17.74	2.90	17.00	2.50	0.26	0.89	0.380	ns
Geometric sequences	18.08	3.32	17.50	3.52	0.17	0.57	0.568	ns
**Total pictorial**	**50.39**	**6.63**	**48.69**	**6.65**	**0.26**	**0.86**	**0.392**	**ns**
**Total geometric**	**53.95**	**6.51**	**52.25**	**6.58**	**0.26**	**0.87**	**0.387**	**ns**
**Total score**	**104.34**	**11.63**	**100.94**	**11.50**	**0.29**	**0.99**	**0.329**	**ns**

F, female (*n* = 38); M, male (*n* = 16); d, Cohen's d. Two-tailed independent-samples *t*-test. ns = *p* > 0.05.

There were no statistically significant gender differences on any subtest or composite. Cohen's d values ranged from 0.09 (Photographic Classification) to 0.29 (Total Score) in this pilot sample, which corresponded to small observed effects. The non-significant results should not be interpreted as confirmation of the absence of gender difference or as confirmation that the instrument is equally effective for boys and girls. A larger and more balanced sample would be needed to establish gender-equivalent functioning, along with formal measurement invariance analyses or differential item functioning analyses. These results are consistent with the culture-fair design intent of the CTONI-2. Additionally, they are similar to the negligible sex-difference pattern found in meta-analysis studies of nonverbal intelligence (Onnivello et al., 2022).

#### 3.4.4 Grade level differences

**Figure 5:**
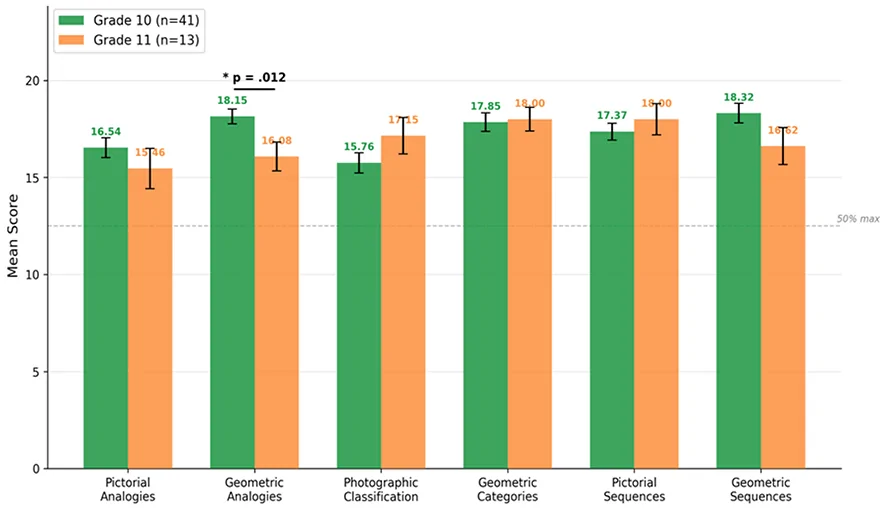
Mean CTONI-2 subtest scores by grade level with data labels. Numbers above bars are group means. Significance bracket (*) marks Geometric Analogies (*p* = 0.012, *d* = 0.83).

Grade 10 and Grade 11 students were compared using independent-samples tests, supplemented by assumption checks, Welch's *t*-tests, Mann–Whitney U tests, and Holm-adjusted *p*-values because of the unequal group sizes. Results are presented in Table 10 and Figure 5.

**Table 10:** Comparison of CTONI-2 scores by grade level.

Subtest	L10 M	L10 SD	L11 M	L11 SD	*d*	*t*	*p*	Sig.
Pictorial analogies	16.54	3.31	15.46	3.76	0.32	0.99	0.328	ns
**Geometric analogies**	**18.15**	**2.45**	**16.08**	**2.69**	**0.83**	**2.60**	**0.012** ^*^	^*^
Photographic classification	15.76	3.34	17.15	3.39	−0.42	−1.31	0.196	ns
Geometric categories	17.85	3.06	18.00	2.20	−0.05	−0.16	0.874	ns
Pictorial sequences	17.37	2.77	18.00	2.89	−0.23	−0.71	0.480	ns
Geometric sequences	18.32	3.27	16.62	3.45	0.51	1.62	0.112	ns
**Total pictorial**	**49.66**	**6.11**	**50.62**	**8.26**	**−0.14**	**−0.45**	**0.654**	**ns**
**Total geometric**	**54.32**	**6.51**	**50.69**	**5.95**	**0.57**	**1.79**	**0.080**	**ns** ^**a**^
**Total score**	**103.98**	**11.09**	**101.31**	**13.33**	**0.23**	**0.72**	**0.475**	**ns**

L10, Level 10 (*n* = 41); L11, Level 11 (*n* = 13); d, Cohen's d. ^a^*p* < 0.10. ^*^p < 0.05.

A grade-level difference was observed for Geometric Analogies, with Grade 10 students (*M* = 18.15, SD = 2.45) scoring higher than Grade 11 students (*M* = 16.08, SD = 2.69), *t*(52) = 2.60, *p* = 0.012, *d* = 0.83. Sensitivity analyses using Welch's t-test and the Mann–Whitney U test produced the same directional pattern, although interpretation was made cautiously after adjustment for multiple comparisons. Total Geometric showed a weaker difference (*p* = 0.080, *d* = 0.57). Given the pilot nature of the adolescent sample and the imbalance between Grade 10 (*n* = 41) and Grade 11 (*n* = 13), these findings should be regarded as exploratory rather than confirmatory. The unexpected direction may reflect cohort imbalance, curricular differences, or sampling variability. No other subtests or composites showed reliable grade-level differences after correction for multiple comparisons.

#### 3.4.5 Age-related performance patterns

**Figure 6:**
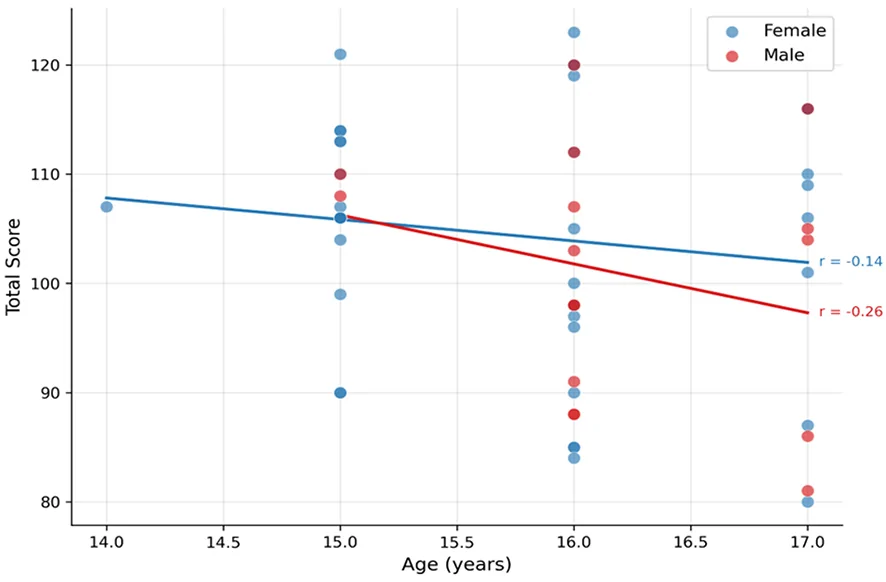
Relationship between age and total CTONI-2 score by gender. Lines = gender-specific OLS regression slopes with Pearson r values. Negative slopes reflect modest age-related decline within this narrow range.

One-way ANOVA results by age group (15, 16, 17 years) and Pearson correlations of age with each score are presented in Table 11 and Figure 6.

**Table 11:** One-way ANOVA results for CTONI-2 scores by age group.

Subtest	Age 15	Age 16	Age 17	*F*	*p*
Pictorial analogies	17.53 (2.74)	15.70 (3.56)	15.46 (3.76)	1.889	0.162
Geometric analogies	18.29 (2.97)	17.96 (2.06)	16.08 (2.69)	3.210	0.049^*^
Photographic classification	15.76 (3.64)	16.35 (3.25)	16.46 (3.31)	0.790	0.459
Geometric categories	18.59 (2.72)	17.57 (2.86)	17.77 (3.19)	1.294	0.283
Pictorial sequences	17.65 (2.29)	17.57 (2.95)	17.31 (3.25)	0.314	0.732
Geometric sequences	18.59 (3.36)	18.00 (3.43)	16.85 (3.44)	1.554	0.221
Total pictorial	51.24 (6.31)	49.35 (5.84)	49.31 (8.08)	0.752	0.476
Total geometric	55.53 (5.57)	52.57 (6.67)	50.23 (5.86)	2.422	0.099
Total score	106.76 (9.61)	101.91 (11.36)	99.54 (13.05)	1.213	0.306

Age 14 with *n* = 1 was excluded from the analysis. Age 15: *n* = 17; Age 16: *n* = 23; Age 17: *n* = 13. Pearson correlations with age: GA *r* = −0.33, *p* = 0.016; GS *r* = −0.28, *p* = 0.040; Total GEO *r* = −0.30, *p* = 0.025. ^*^p < 0.05.

Pearson correlations revealed statistically significant negative associations between age and Geometric Analogies (*r* = −0.33, *p* = 0.016), Geometric Sequences (*r* = −0.28, *p* = 0.040), and Total Geometric (*r* = −0.30, *p* = 0.025). Pictorial subtests showed no significant age relationships. One-way ANOVA confirmed a significant age-group effect for Geometric Analogies alone, *F*(2, 50) = 3.21, *p* = 0.049, with means declining monotonically from age 15 (*M* = 18.29) to age 17 (*M* = 16.08). Given that older students are enrolled in the more advanced grade (11), this pattern likely reflects the same grade-level effect identified in Section 3.4.4 rather than a true developmental decline—cross-sectional designs within narrow age bands are susceptible to cohort confounds (Moore et al., 2017).

#### 3.4.6 Intercorrelation structure

**Figure 7:**
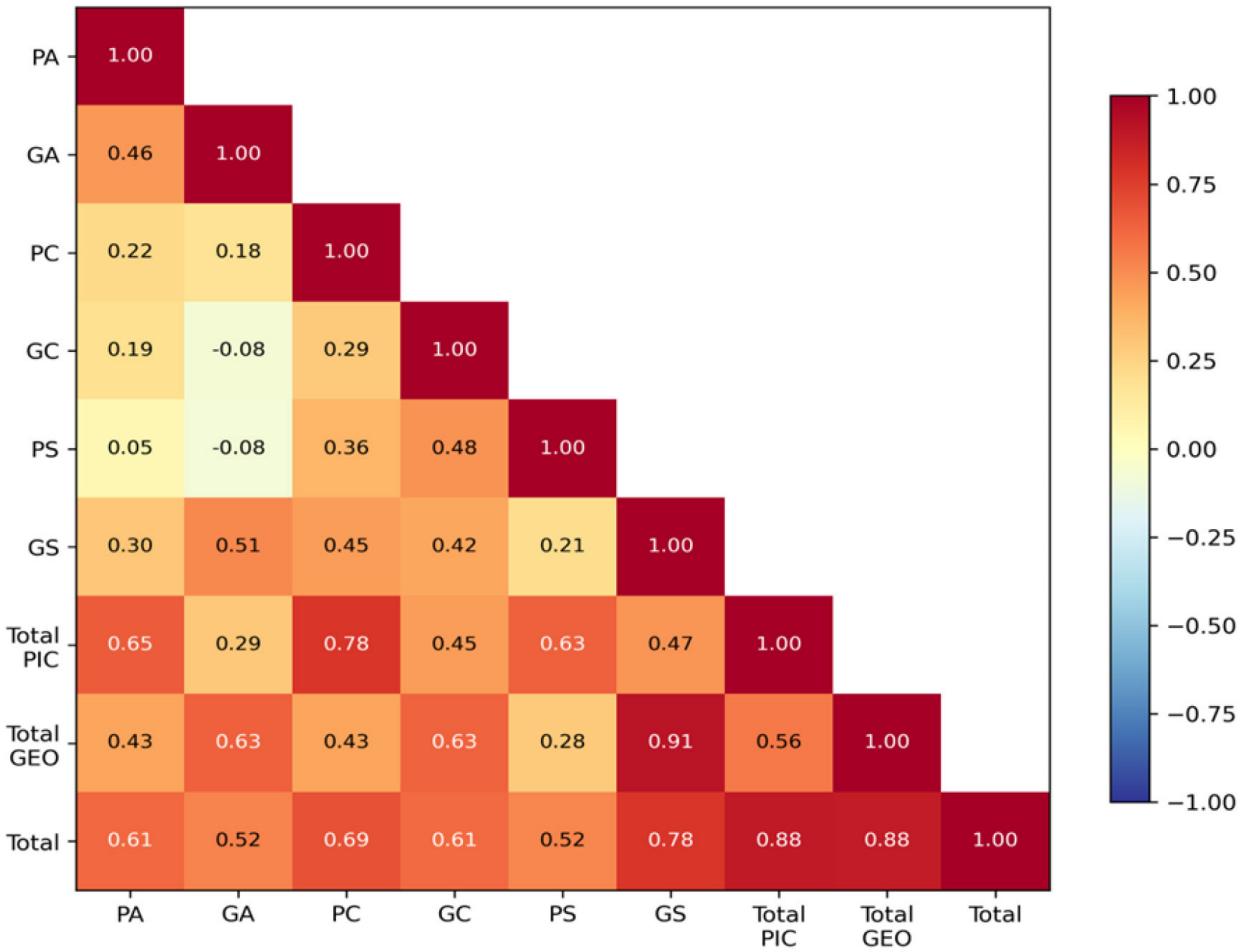
Intercorrelation heat map for CTONI-2 subtests and composite scores. Color scale: dark red = strong positive correlation (approaching 1.0); light = weak/negative. Lower triangle values shown.

Pearson intercorrelations among subtests and composites are presented in Table 12 and Figure 7.

**Table 12:** Intercorrelation matrix of CTONI-2 subtests and composite scores (*N* = 54).

Subtest	PA	GA	PC	GC	PS	GS	PIC	GEO	Total
PA	1.00	0.46^**^	0.22	0.19	0.05	0.30^*^	0.65^**^	0.43^**^	0.61^**^
GA		1.00	0.18	−0.08	−0.08	0.51^**^	0.29^*^	0.64^**^	0.52^**^
PC			1.00	0.29^*^	0.36^**^	0.45^**^	0.78^**^	0.43^**^	0.69^**^
GC				1.00	0.48^**^	0.42^**^	0.45^**^	0.63^**^	0.61^**^
PS					1.00	0.21	0.63^**^	0.28^*^	0.52^**^
GS						1.00	0.47^**^	0.91^**^	0.78^**^
PIC							1.00	0.56^**^	0.89^**^
GEO								1.00	0.88^**^
Total									1.00

PA, pictorial analogies; GA, geometric analogies; PC, photographic classification; GC, geometric categories; PS, pictorial sequences; GS, geometric sequences. ^*^p < 0.05. ^**^p < 0.01.

Geometric Sequences showed the highest correlation with Total Geometric (*r* = 0.91) and was effectively a proxy for the composite. Within the PA–PC (*r* = 0.22), PA–PS (*r* = 0.05), and PC–PS correlations, the strongest were found between the pairs within the same domain, and the between-domain correlations were more variable. These two composites also had significant correlation with Total Score (*r* = 0.89 and 0.88, respectively) which verified the one-dimensionality that was confirmed by CFA. The moderate between domain correlations (Total PIC–Total GEO: *r* = 0.56) are similar to the level of partial separability of Pictorial and Geometric reasoning as reported during standardization.

## 4 Discussion

Several theoretical and practical contributions are made in the psychometric adaptation of the CTONI-2 for Turkish children. The moderate CFA loadings (range 0.55–0.64) and acceptable composite reliability (CR = 0.78) are consistent with the broad-construct nature of nonverbal intelligence: although the six subtests share a common latent factor, each also reflects unique domain-specific variance that appropriately attenuates the AVE below 0.50. This is not a deficiency but an expected consequence of sampling across two content domains rather than within one. Interestingly, the test–retest coefficients obtained here (0.81–0.96) are comparable to the intra-class correlation reported for the TONI-4 in a clinical schizophrenia population (Chen et al., 2021), which is a desirable test–retest stability related to the instruments' ability to capture cognitive changes over time.

The secondary-school application findings should be interpreted as preliminary descriptive evidence. The observed raw-score profile suggested that students in this small pilot sample obtained scores across a usable range, with no clear floor or ceiling concentration. However, because the sample was small and was not designed for adolescent norming, these results should not be interpreted as evidence of normative adequacy or full sensitivity across the ability range. The Total Geometric score was numerically higher than the Total Pictorial score in this sample, but this pattern was not supported by direct assessment of curriculum exposure, mathematics achievement, or a larger representative adolescent sample. Therefore, the geometric-pictorial difference is presented only as a descriptive pattern that requires further investigation. Similarly, It is reported from the mathematics education in East Asian and Middle Eastern countries where geometry is a more emphasized part of secondary school curriculum (Summaka et al., 2024).

The finding of no gender differences is in line with the consensus found in meta-analyses (Onnivello et al., 2022) and with the culture-fair design of the CTONI-2. The advantage for Grade 10 in Geometric Analogies (*d* = 0.83) and the negative correlation between age and geometric reasoning in the 14–17 age span should be interpreted as tentative pilot findings. The small and imbalanced grade distribution, particularly the Grade 11 group (*n* = 13), may have contributed to these results. Larger and more balanced adolescent samples are needed before drawing conclusions about grade-level or age-related differences.

The Turkish CTONI-2 fills a specific gap in the Turkish assessment landscape by providing initial reliability and validity evidence for a language-reduced nonverbal reasoning measure in primary-school children. Because the CTONI-2 reduces verbal demands, future research may examine its psychometric performance in populations whose assessment can be influenced by language or communication-related factors, including hearing-impaired students (Hasnain et al., 2023), learners with communication disorders or autism-related communication difficulties (Berube, 2021), twice-exceptional learners (Alameddine, 2025), and recently arrived immigrant or asylum-seeking students with limited host-language proficiency (Rogers and Tazi, 2025). These populations were not included in the present study, and no conclusions can be drawn regarding the suitability, fairness, or diagnostic utility of the Turkish CTONI-2 for these groups based on the current evidence.

Similarly, future studies should evaluate the psychometric performance and practical applicability of the Turkish CTONI-2 in clinical and forensic settings before its use can be recommended in these contexts (Romero-Martínez et al., 2026). Such investigations should include evidence of measurement invariance, differential item functioning, and validity within the target populations.

### 4.1 Limitations and future directions

Despite its contributions, this study has several limitations. First, the AVE value for the single-factor CFA model was below the conventional 0.50 criterion, indicating limited average variance explained by the general nonverbal intelligence factor across the six subtests. Although Composite Reliability was acceptable, the low AVE suggests that subtest-specific variance remains meaningful and that the evidence for convergent validity should be interpreted cautiously. Future research should test alternative models, including correlated pictorial and geometric factors, and should evaluate whether the factor structure is stable across age groups. The adaptation and validation stage was also restricted to primary-school children from two schools in Ankara, and the adolescent component was based on a small pilot application sample that was not designed to establish Turkish age-based norms. In the adolescent application stage, score interpretation relied on the original CTONI-2 normative tables reported by Hammill et al. (2009), because Turkish adolescent norms were not available. Therefore, the adolescent findings should be interpreted as preliminary evidence of applied score profiles rather than as evidence of full cultural standardization for adolescents. The small and uneven grade distribution in this sample, particularly the imbalance between Grade 10 (*n* = 41) and Grade 11 (*n* = 13), may also have contributed to the atypical negative correlations between age and performance on the geometric subtests. Further studies with larger, regionally diverse, balanced, and age-stratified Turkish adolescent samples are needed before normative, grade-related, or age-related claims can be made for secondary-school students. In addition, the psychometric behavior of the instrument was not examined in special populations for whom language-reduced assessment may be particularly relevant, including hearing-impaired learners, students on the autism spectrum, twice-exceptional learners, and recently arrived immigrant students. No clinical sample was included for criterion validity analysis. Finally, because of the cross-sectional design, developmental trends could not be separated from grade-level cohort effects in the age and geometric reasoning relationship.

Three directions are suggested for future research. First, to support individual level clinical decisions, Turkish norms representative of the Turkish population need to be established for the entire age range of 6–18 years. Second, measurement invariance studies across special populations are necessary to ensure equitable applicability, especially for special populations on which language-free assessment will have the most impact. Third, longitudinal studies that examine the performance of CTONI-2 in relation to academic success and verbal intelligence would help to elucidate the incremental validity of the nonverbal test in the Turkish school setting and provide more evidence for its use in the regular educational psychology practice.

## 5 Conclusion

This study provides initial evidence supporting the reliability and validity of the Turkish adaptation of the CTONI-2 for primary-school children aged 8 to 10 years. The adaptation preserved the conceptual framework of the original instrument while incorporating culturally appropriate modifications to selected pictorial items, providing a language-reduced measure of nonverbal reasoning for educational assessment and research in the Turkish context.

The secondary-school application was conducted as an exploratory pilot to examine score patterns in an older age group rather than to establish adolescent norms. Accordingly, these findings should be interpreted cautiously because of the small and unevenly distributed pilot sample and should not be generalized beyond the present study.

The findings provide initial evidence for the reliability and validity of the Turkish CTONI-2 in the primary-school adaptation sample and preliminary descriptive application evidence in a small secondary-school pilot sample. Although the language-reduced nature of the CTONI-2 may make it potentially relevant for learners whose assessment performance is influenced by language or communication-related factors (Hasnain et al., 2023; Berube, 2021; Alameddine, 2025; Rogers and Tazi, 2025), these populations were not included in the present study, and no conclusions can be drawn regarding the suitability or fairness of the Turkish CTONI-2 for these groups. Future research should establish representative Turkish norms across broader age groups, evaluate measurement invariance and differential item functioning, and examine the psychometric performance of the Turkish CTONI-2 in larger and more diverse educational and clinical populations.

## Data Availability

The raw data supporting the conclusions of this article will be made available by the authors upon reasonable request to the corresponding author, without undue reservation.
